# Supplementary material for: Degradation of engulfed mitochondria is rate-limiting in Optineurin-mediated mitophagy in neurons
Source: eLife. 2020 Jan 14;9:e50260. doi: 10.7554/eLife.50260 (PMC6959996; doi:10.7554/eLife.50260)
Supplement: Supplementary file 1. — A list of key reagents in this study, including reagent type, designation, source, and identifier (when available or applicable). [file elife-50260-supp1.docx]

| Key Resources Table | | | | |
| --- | --- | --- | --- | --- |
| **Reagent type (species) or resource** | **Designation** | **Source or reference** | **Identifiers** | **Additional information** |
| Strain (*Rattus norvegicus*) | Sprague Dawley | Charles River Laboratories |  |  |
| Cell line (*Homo-sapiens*) | HeLa-M | A. Peden (Cambridge Institute for Medical Research) |  | Authenticated by STR profiling; tested negative for mycoplasma |
| Transfected construct (*Homo-sapiens*) | Mito-DsRed | Clontech | Cat # 632421 | Provided by T. Schwartz (Harvard Medical School) |
| Transfected construct (*Homo-sapiens*) | Mito-SNAP | PMID:27484150 |  | Subcloned from Mito-DsRed into RRID:Addgene_58187 |
| Transfected construct (*Homo-sapiens*) | Cox8-EGFP-mCherry | This paper |  | Subcloned from RRID:Addgene_78520 |
| Transfected construct (*Rattus norvegicus*) | EGFP-LC3B | Addgene; PMID:11060023 | RRID:Addgene_21073 | Provided by T. Yoshimori (Osaka University) |
| Transfected construct (*Rattus norvegicus*) | mScarlet-LC3B | This paper |  | Subcloned from RRID:Addgene_21073; EGFP was replaced with mScarlet from RRID:Addgene_85054 |
| Transfected construct (*Homo-sapiens*) | mCherry-GFP-LC3 | Addgene; PMID:22331844 | RRID:Addgene_123230 | Provided by T. Johansen (University of Tromso) |
| Transfected construct (*Homo-sapiens*) | EGFP-OPTN | PMID:2167041 |  | Provided by I. Dikic (Goethe University) |
| Transfected construct (*Homo-sapiens*) | Halo-OPTN | PMID:27484150 |  | Subcloned from EGFP-OPTN into pHaloTag vector (Promega Cat # G7721) |
| Transfected construct (*Homo-sapiens*) | Halo-OPTN^E478G^ | PMID:27484150; PMID:2167041 |  | Subcloned into pHaloTag vector (Promega Cat # G7721) from pcDNA3.1 (+)/HA-OPTN E478G provided by I. Dikic (Goethe University) |
| Transfected construct (*Homo-sapiens*) | Untagged-Parkin | PMID:2724738; PMID:25294927 |  | The mCherry from RRID:Addgene_23956 was removed |
| Transfected construct (*Homo-sapiens*) | YFP-Parkin | Addgene; PMID:19029340 | RRID:Addgene_23955 | Provided by R. Youle |
| Transfected construct (*Homo-sapiens*) | mCherry-Parkin | Addgene; PMID:19029340 | RRID:Addgene_23956 | Provided by R. Youle |
| Transfected construct (*Homo-sapiens*) | mCherry-Parkin^T240R^ | PMID:25294927 |  | Generated by site-directed mutagenesis from mCherry-Parkin |
| Transfected construct (*Homo-sapiens*) | GFP-Ub | Addgene; PMID:16606690 | RRID:Addgene_11928 |  |
| Transfected construct (*Rattus- norvegicus*) | YFP-NaV_II-III_ | Addgene; PMID:20543823 | RRID:Addgene_26056 |  |
| Transfected construct (*Homo-sapiens*) | SNAP-TBK1 | PMID:27484150 |  | Subcloned from RRID:Addgene_23851 into RRID:Addgene_58186 |
| Transfected construct (*Rattus- norvegicus*) | LAMP1-EGFP | This Paper |  | Subcloned from RRID:Addgene_1817 where RFP was replaced with EGFP |
| Transfected construct (*Rattus- norvegicus*) | SEP-LAMP1-RFP | PMID:28320970 |  | Provided by J. Bonifacino (NIH) |
| Antibody | Anti-Parkin (PRK8) (Mouse Monoclonal) | Santa Cruz | Cat # sc-32282; RRID:AB_628104 | WB (1:100) |
| Antibody | Anti-TBK1/NAK (Rabbit Monoclonal) | Abcam | Cat # ab40676; RRID:AB_776632 | WB (1:1000) |
| Antibody | Anti-Optineurin (Rabbit Polyclonal) | ThermoFisher | Cat # 711879;  RRID:AB_2723433 | WB (1:100) |
| Antibody | Anti-OPTN (Rabbit Polyclonal) | Cayman Chemical | Cat # 100000; RRID:AB_327788) | ICC (1:50) |
| Antibody | Anti-LAMP1 (Rabbit Polyclonal) | Abcam | Cat # ab24170; RRID:AB_775978 | WB (1:250) |
| Antibody | Anti-LC3B (Rabbit Polyclonal) | Abcam | Cat # ab48394; RRID:AB_881433 | WB (1:1000) |
| Antibody | Anti-p62/SQSTM1 (Mouse Monoclonal) | Abcam | Cat # ab56416; RRID:AB_945626 | WB (1:1000), ICC (1:100) |
| Antibody | Anti-TRAF6BP/TAX1BP1 (Rabbit Monoclonal) | Abcam | Cat # ab176572 | WB (1:1000) |
| Antibody | Anti-NDP52 (Rabbit Polyclonal) | Novus Biologicals | Cat # NBP2-19499 | WB (1:500) |
| Antibody | Anti-ATG16L1 (Rabbit Monoclonal) | Abcam | Cat # ab187671 | WB (1:1000) |
| Antibody | Anti-ATG5 (Chicken Polyclonal) | Novus Biologicals | Cat # NBP1-76992; RRID:AB_11033378 | WB (1:250) |
| Antibody | Anti-Neurofilament H (NF-H) (Mouse Monoclonal) | BioLegend | Cat # 801601; RRID:AB_2564641 | ICC (1:2000) |
| Antibody | Anti-Tau (Rabbit Polyclonal) | Abcam | Cat # ab8763 | ICC (1:3000) |
| Antibody | Anti-Microtubule-Associated Protein (MAP2) (Chicken Polyclonal) | Aves | Cat # MAP | ICC (1:2000) |
| Antibody | Anti-TFEB (Goat Polyclonal) | Abcam | Cat # ab2636; RRID:AB_303224 | WB (1:100) |
| Antibody | Anti-Phospho-TFEB (Ser211) (Rabbit Monoclonal) | Cell Signaling | Cat # 37681; RRID:AB_2799117 | WB (1:250) |
| Antibody | Anti-Cathepsin D (Rabbit Polyclonal) | Novus Biologicals | Cat # NBP1-50682; RRID:AB_11019358 | WB (1:250) |
| Antibody | Anti-Chicken IgY-AlexaFluor488 (Goat Polyclonal) | ThermoFisher | Cat # A011039; RRID:AB_142924 | ICC (1:500) |
| Antibody | Anti-Mouse IgG-AlexaFluor546 (Donkey Polyclonal) | ThermoFisher | Cat # A-10036; RRID:AB_2534012 | ICC (1:500) |
| Antibody | Anti-Rabbit IgG-AlexaFluor647 (Donkey Polyclonal) | ThermoFisher | Cat # A-31573; RRID:AB_2536183 | ICC (1:500) |
| Antibody | Anti-GoatIgG-AlexaFluor680 (Donkey Polyclonal) | ThermoFisher | Cat # A-21084; RRID:AB_141494 | WB (1:20,000) |
| Antibody | Anti-Mouse IgG-IRDye 800CW (Donkey Polyclonal) | LI-COR | Cat # 926-32212; RRID:AB_621847 | WB (1:20,000) |
| Antibody | Anti-Rabbit IgG-IRDye 800CW (Donkey Polyclonal) | LI-COR | Cat # 926-32213; RRID:AB_621848 | WB (1:20,000) |
| Antibody | Anti-Rabbit IgG-IRDye 680RD (Donkey Polyclonal) | LI-COR | Cat # 926-68073; RRID:AB_10954442 | WB (1:20,000) |
| Antibody | Anti-Chicken IgG-IRDye 680RD (Donkey Polyclonal) | LI-COR | Cat # 926-68075; RRID:AB_10974977 | WB (1:20,000) |
| Sequence-based reagent | ON-TARGET*Plus* Rat OPTN (246294) siRNA *SMARTpoo*l | Dharmacon | Cat # L-097177-02-0005 | Proprietary sequence |
| Sequence-based reagent | ON-TARGET*Plus* Rat Prkn (56816) siRNA *SMARTpoo*l | Dharmacon | Cat # L-090709-02-0005 | Proprietary sequence |
| Chemical compound, drug | JF646 HaloTag | Promega | Cat # GA1120 |  |
| Chemical compound, drug | JF646-SNAP | Luke Lavis, Janelia Farms (HHMI) |  |  |
| Chemical compound, drug | SNAP-Cell Block | New England Biolabs | Cat # S9106S |  |
| Chemical compound, drug | SNAP-Cell 430 | New England Biolabs | Cat # S9109S |  |
| Chemical compound, drug | SNAP-Cell TMR-Star | New England Biolabs | Cat # S9015S |  |
| Chemical compound, drug | CellROX Deep Red Reagent | Invitrogen | Cat # C10422 |  |
| Chemical compound, drug | B-27 Supplement (50X), minus antioxidants | ThermoFisher | Cat # 10889038 |  |
| Chemical compound, drug | LysoT (LysoTracker Green DND-26) | ThermoFisher | Cat # L7526 |  |
| Chemical compound, drug | TMRE (tetramethylrhodamine ethyl ester, Ethyl Ester, Perchlorate | Life Technologies | Cat # T-669 |  |
| Software, algorithm | Leica Application Suite (LAS) X | Leica Microsystems |  |  |
| Software, algorithm | Volocity | PerkinElmer |  |  |
| Software, algorithm | FIJI | PMID:22743772 |  |  |
| Software, algorithm | Prism 6, Prism 8 | GraphPad |  |  |
| Software, algorithm | Adobe Illustrator CC 2018 and 2019 | Adobe Systems |  |  |
| Software, algorithm | Image Studio | LI-COR |  |  |
